# A systematic review of prediction models to diagnose COVID-19 in adults admitted to healthcare centers

**DOI:** 10.1186/s13690-021-00630-3

**Published:** 2021-06-18

**Authors:** Médéa Locquet, Anh Nguyet Diep, Charlotte Beaudart, Nadia Dardenne, Christian Brabant, Olivier Bruyère, Anne-Françoise Donneau

**Affiliations:** 1 WHO Collaborating Centre for Public Health, Aspects of Musculo-Skeletal Health and Ageing, Research Unit in Public Health, Epidemiology and Health, Economics, University of Liège, Quartier Hôpital, Av. Hippocrate 13, CHU B23, 4000 Liège, Belgium; 2 Biostatistics Unit, University of Liège, Quartier Hôpital, Av. Hippocrate 13, CHU B23, 4000 Liège, Belgium

**Keywords:** COVID-19, Prediction model, Hospitalisation

## Abstract

**Background:**

The COVID-19 pandemic is putting significant pressure on the hospital system. To help clinicians in the rapid triage of patients at high risk of COVID-19 while waiting for RT-PCR results, different diagnostic prediction models have been developed. Our objective is to identify, compare, and evaluate performances of prediction models for the diagnosis of COVID-19 in adult patients in a health care setting.

**Methods:**

A search for relevant references has been conducted on the MEDLINE and Scopus databases. Rigorous eligibility criteria have been established (e.g., adult participants, suspicion of COVID-19, medical setting) and applied by two independent investigators to identify suitable studies at 2 different stages: (1) titles and abstracts screening and (2) full-texts screening. Risk of bias (RoB) has been assessed using the Prediction model study Risk of Bias Assessment Tool (PROBAST). Data synthesis has been presented according to a narrative report of findings.

**Results:**

Out of the 2334 references identified by the literature search, 13 articles have been included in our systematic review. The studies, carried out all over the world, were performed in 2020. The included articles proposed a model developed using different methods, namely, logistic regression, score, machine learning, XGBoost. All the included models performed well to discriminate adults at high risks of presenting COVID-19 (all area under the ROC curve (AUROC) > 0.500). The best AUROC was observed for the model of Kurstjens et al (AUROC = 0.940 (0.910–0.960), which was also the model that achieved the highest sensitivity (98%). RoB was evaluated as low in general.

**Conclusion:**

Thirteen models have been developed since the start of the pandemic in order to diagnose COVID-19 in suspected patients from health care centers. All these models are effective, to varying degrees, in identifying whether patients were at high risk of having COVID-19.

**Supplementary Information:**

The online version contains supplementary material available at 10.1186/s13690-021-00630-3.

## Introduction

Coronavirus disease 2019 (COVID-19) is an emerging disease linked to a new virus, the Severe Acute Respiratory Syndrome CoronaVirus 2 (SARS-CoV-2), which quickly spreads across the world and is now responsible for a global pandemic. The public health impact of the disease is considerable. More than 102 million cases of infections are currently recorded worldwide, with hospitalization rates reaching 18% depending on the age groups [1], leading to more than 2 million deaths [2] and to significant incremental costs to the healthcare system. In addition, collateral damage through an increase in non-communicable diseases, dramatically increases the public health burden associated with COVID-19 [3]. It is, therefore, a public health emergency, as declared by the World Health Organization. In view of these findings, it is obvious that there is significant pressure on hospital health system resources. However, hospitals are actually filled with confirmed COVID-19 patients who need intensive medical care but also with suspected COVID-19 cases who do not necessarily require to be hospitalized. It is therefore essential to quickly and efficiently determine whether a patient is likely to be positive for COVID-19 right after being admitted in a healthcare center to optimize triage and limit the risk of infections contracted inside the hospital. During the first wave, 12.5% of COVID-19 cases observed in British and Italian hospitals have happened to be nosocomial infections [4].

Despite a certain rate of false-negative results [5], the gold standard is the real-time Reverse Transcription-Polymerase Chain Reaction (RT-PCR) test for etiological diagnosis of COVID-19 cases. However, the estimated time to obtain the results of this test is often more than 24 h due to laboratory work overload, making direct triage of patients at high risk of infection difficult. COVID-19 history, clinical judgment and routine clinical investigations (e.g., such as laboratory data and radiological images) could therefore prove to be of great help to promptly identify patients at high risk of suffering from COVID-19.

Different diagnostic prediction models have been published in the scientific literature. According to Wynants et al. [6], there are some 33 diagnostic models for predicting COVID-19. Among these, only the model by Jehi et al. was identified as promising to be further validated. Other models were assessed as poorly reported with high or unclear Risk of Bias (RoB) in terms of the representativeness of population and lack of external or internal validation. However, according to Brown et al. [7] and subsequently confirmed by Wynants et al. [6], an overall RoB was assessed rather than the applicability of the predictors because the review aimed to document all the available COVID-19 related prediction models, not to answer specific review questions.

In order to evaluate the extent to which COVID-19 prediction models can be applicable in clinical practice, the present systematic review is designed with a more focused research question. Our objective is, therefore, to carry out a systematic review of the most recent literature to identify, evaluate and compare existing models that could prove to be of added value for clinicians in a hospital setting to sort and categorize adult patients at high risk of COVID-19.

## Methods

To ensure quality and transparence of reporting in this systematic review, we followed the Preferred Reporting Items for Systematic Reviews and Meta-analyses (PRISMA) guidelines [8]. A protocol has been previously registered at the PROSPERO International Prospective Register of Systematic Reviews website (under the reference CRD42021230975). Our issue of interest was first identified and defined using the following PICOS strategy: Population - All participants aged 18 years old and over, with suspected COVID-19 infection in health care settings; Intervention/Exposure – Participants have to be evaluated for COVID-19 infection; Comparator - Not applicable; Outcome – diagnosis of COVID-19 using RT-PCR; Study design – Diagnostic prediction models. Our goal was therefore to systematically search and summarize the studies presenting diagnostic models of COVID-19 in adults with suspected COVID-19 infection in health care settings.

### Literature search strategies

The search began on January 4, 2021, applying a specific search strategy on MEDLINE (via Ovid) and Scopus (via Elsevier). Additionally, we manually searched the reference list of the included studies and Google Scholar search engine. An update has been carried out on February 26, 2021. The search strategy consisted of two key concepts: (1) COVID-19 and (2) prediction models for diagnosis. The whole search strategy is available in Supplementary Material.

### Inclusion and exclusion criteria

We developed prespecified eligibility criteria to determine the inclusion of abstracts and articles. Briefly, we included peer-reviewed original studies presenting prediction models for diagnosis of COVID-19 in adults from health care departments. The prognostic models aiming to evaluate the evolution of the COVID-19, and thus not meeting our research objective, were excluded. English language was required (restriction not impacting the quality of the systematic review [9]), and the publication date considered was from May 1, 2020 to January 4, 2021, with an update performed on February 26, 2021. Published studies meeting our inclusion criteria before May 1, 2020 were identified by means of a previously published systematic review of Wynants et al. in the British Medical Journal because it partially covering our topic [6]. Table 1 provides the whole inclusion and exclusion criteria applied to meet our objective.

**Table 1:** Eligibility criteria of references to be included in the systematic review of prediction models to diagnose COVID-19 from the start of the epidemic to March 2021

Inclusion criteria	Exclusion criteria
Prediction model studies for diagnosis of COVID-19 Peer-reviewed original studies Population included patients with suspected infection, aged 18 years old and over Medical setting English language Publication date from May 1st 2020 to January 4th 2021	Studies without primary data Studies which developed a prognostic model Studies presenting epidemics modelling Ecological studies Interventional studies Studies with a disease-specific sample Preprint articles Review articles Protocols

### Selection of studies

Titles and abstracts of references identified by the search strategy were independently reviewed by 3 reviewers (AD, CB and ML) according to the aforementioned eligibility criteria. The full-text review stage followed, and eligible studies were identified by 2 reviewers (AD and ML). At each stage, if disagreements occurred, they were resolved by discussion between reviewers with the intervention of a third peer (CB) if needed, to arbitrate in final inclusion. This entire procedure was performed using the covidence® systematic review management software recommended by Cochrane collaboration.

### Data extraction

A standardized data extraction form has been developed. A pre-pilot extraction of a first reference was carried out to assess the relevance of the data extraction form. Then, relevant data were extracted independently by 2 reviewers (AD and ML) and discrepancies were resolved through discussion with the help of a third peer (CB) if necessary. The extraction has been performed according to the CHecklist for critical Appraisal and data extraction for systematic Reviews of prediction Modelling Studies (CHARMS) [10]. Different data of interest have thus been collected: manuscript general information, population description, characterization of predictors and outcome, model development, model performance, results and conclusions.

### Risk of bias

Two reviewers (AD and ML) assessed the quality and RoB of each included study using the Prediction model study Risk Of Bias Assessment Tool (PROBAST) [11], designed specifically for systematic reviews of diagnostic prediction models. The RoB has been assessed through questions regarding several domains: participants, predictors, outcome, analysis. The questions were answered by “high risk of bias”, “unclear risk of bias”, “low risk of bias”. Any conflict has been resolved through consensus, optionally with the intervention of a third party (CB).

### Data synthesis

A descriptive analysis of the included studies was performed under the format of a narrative report. Results were structured according to a primary description of the general characteristics of the included studies, followed by the model development and model performance to conclude with a comprehensive description of the final model.

## Results

**Fig. 1:**
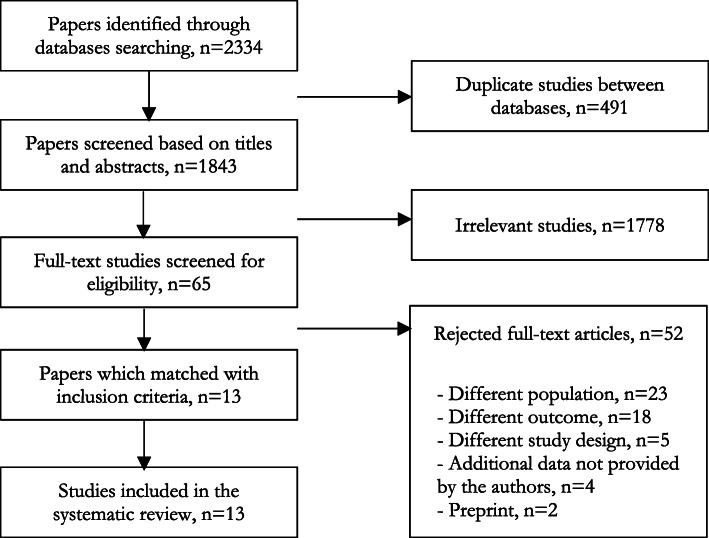
PRISMA flow diagram for the inclusion of prediction models to diagnose COVID-19 developed during the pandemic until March 2021

Of 1843 abstracts reviewed, we identified 65 articles for full-text screening. Subsequently, 13 of these met eligibility criteria as shown in Fig. 1 [12–24].

Supplementary material offers a listing of all excluded refences (*n* = 52) at the full-text screening stage, as recommended by AMSTAR2 [25].

The general characteristics of each included study are described in Table 2. All the studies were performed from 2020, and all around the world.

**Table 2:** General characteristics of the included studies in the systematic review of prediction models to diagnose COVID-19 from the start of the epidemic to March 2021

Reference	Year of publication	Country	Medical setting
Aldobyany [12]	2020	Saudi Arabia	Tertiary care facility
Bar [13]	2020	China	Emergency room
Callejon-Leblic [17]	2021	Spain	General hospital
Fink [18]	2020	UK	Secondary care
Gupta-Wright [19]	2020	UK	General hospital
Huang [20]	2020	China	COVID-19 hospital
Kurstjens [21]	2020	Netherlands	Emergency room
McDonald [22]	2020	USA	Quaternary care
Nakakubo [23]	2020	Japan	General hospital
Plante [24]	2020	USA	Emergency room
Sung [16]	2020	USA	Emergency room
Tordjman [14]	2020	France	Emergency room
Vieceli [15]	2020	Brazil	Hospitalized patients

Each study proposed diagnostic models for COVID-19 based on socio-demographics, clinical symptoms, blood tests, or other characteristics which were compared to the gold standard diagnosis, namely the RT-PCR test. A detailed presentation of the different models developed is available in Table 3. The population consisted of adults’ patients who presented to hospital because of suspicion of COVID-19.

**Table 3:** Description of the different diagnostic models for COVID-19 in the systematic review of prediction models to diagnose COVID-19 from the start of the epidemic to March 2021

Reference	Population	Gold standard	Number of candidate predictors	Type of predictors	Sample size	Modeling method	Final model
Aldobyany [12]	All patients screened for COVID-19 at a tertiary care facility	RT-PCR test	11	Exposure risk Clinical signs Symptoms Clinical history	1435	Score Logistic regression	Exposure risk: A history of travel abroad during the 14 days prior to symptoms onset. OR Visiting or being a resident of a high-risk area for COVID-19 in the kingdom during the 14 days prior to symptom onset. OR A close physical contact with a confirmed case of COVID-19 or MERS-COV in the past 14 days. OR An exposure to camel or camel products in the past 14 days. OR Working in a healthcare facility = 3 points Fever or recent history of fever = 2 points Cough = 2 points Shortness of breath = 2 points Nausea, vomiting, and/or diarrhea = 1 point Chronic renal failure, CAD/heart failure, Immunocompromised patient = 1 point Maximum = 11 points Discriminative score: 4 points or 5 points Multivariate analysis (not used as a model) Sex (male): OR = 1.47 (1.03–2.09), *p* = 0.034 Healthcare workers and their family members: OR = 1.99 (1.14–3.50), *p* = 0.016 Fever: OR: 2.98 (1.97–4.5), *p* < 0.001 Moderate disease severity: OR = 5 (1.23–20), *p* < 0.001
Bar [13]	Adults admitted to emergency room	RT-PCR test	21	Clinical data Imaging Laboratory test	100	Logistic regression	Logistic regression: Intercept: β = − 1.95 qSOFA score > 1: β = 0.05; OR = 1.05 (1.01–1.10) Upper sites B-lines ≥3: β = 0.42; OR = 1.52 (1.31–1.79) Lower sites thickened pleura: β = 0.55; OR = 1.73 (1.49–1.98) Lower sites consolidation: β = 0.87; OR = 2.39 (2.07–2.69) Posterolateral sites thickened pleura: β = 0.68; OR = 1.97 (1.72–2.22)
Callejon-Leblic [17]	Individuals aged 18 or older that were suspected of having COVID-19	RT-PCR test	11	Demographics Clinical data Symptoms	777	Logistic regression Machine learning	x = − 1.76 + 0.88 × ((1 if VAS for loss of smell ≥21) or (0 if VAS for loss of smell < 21)) + 1.83 × ((1 if VAS for loss of taste ≥44) or (0 if VAS for loss of taste < 44)) + 0.79 × ((1 if VAS for dyspnea ≥28) or (0 if VAS for dyspnea < 28)) + 0.61 × ((1 if fever) or (0 if no fever)) + 0.70 × ((1 if diarrhea) or (0 if no diarrhea)) − 1.13 × ((1 if female) or (0 if male)) Loss of smell: OR (95%CI) = 2.42 (1.3–4.5), *p* = 0.0053 Loss of taste: OR (95%CI) = 6.21 (3.21–12.04), p < 0.001 Dyspnea: OR (95%CI) = 2.21 (1.34–3.64), *p* = 0.002 Fever: OR (95%CI) = 1.84 (1.18–2.87), *p* = 0.007 Diarrhea: OR (95%CI) = 2.02 (1.29–3.16), *p* = 0.002 Sex (Male): OR (95%CI) = 3.11 (1.97–4.90), p < 0.001
Fink [18]	Patients’ age > 18 years admitted to hospital for minimum 24 h	RT-PCR test	9	Demographics Symptoms Laboratory test	581	Logistic regression	Final logistic regression: Age: β = 0.018; SE = 0.008, z = 2.18, *P* > z = 0.029, 95%CI = 0.001–0.033 Fever: β = 1.547; SE = 0.2931, z = 5.28, *P* > z = 0.000, 95%CI = 0.973–2.122 Maximal FiO2: β = 0.022; SE = 0.007, z = 2.94, *P* > z = 0.003, 95%CI = 0.007–0.0372 CRP: β = 0.0045; SE = 0.0016, z = 2.174 *P* > z = 0.006, 95%CI = 0.001–0.0078 Normal chest X-ray: β = − 1.113497; SE = 0.2879424, z = − 3.87, *P* > z = 0.000, 95%CI = − 1.677854- -0.5491403 Neutrophiles: β = − 0.1611107; SE = 0.0321778, z = − 5.01, *P* > z = 0.000, 95%CI = − 0.2241779- -0.0980434 Intercept: β = − 0.0367507; SE = 0.6817362, z = − 0.05, *P* > z = 0.957, 95%CI = − 1.372929-1.299428
Gupta-Wright [19]	Patients were included in this study if they were admitted via the acute medical team between 2 March and 3 May 2020 inclusive.	RT-PCR test	15	Demographics Comorbidities Symptoms Laboratory test	2940	Logistic regression Score	Intercept = −4.0 (− 4.4 - -3.6) Age (50–70): β = 0.53 (0–0.41); Point = 1 Sex (Male): β = 0.23 (0.3–0.73); Point = 1 Ethnicity - Asian: 0.6 (0.4–0.82); Point =1 Ethnicity - Black: 0.62 (0.3–0.93); Point = 1 Ethnicity - Mixed/other: 0.81 (0.4–1.2); Point = 1 Ethnicity - Unknown: 0.57 (0.3–0.85); Point = 1 Cough, fever or shortness of breath: 1.13 (0.9–1.35); Point = 1 National Early Warning Score 2 (NEWS) > 5: 0.87 (0.7–1.05); Point = 2 CRP > 50: 1.13 (1–1.32); Point = 2 Lymphocytes < 1: 0.54 (0.4–0.73); Point = 1 Chest X-ray - Lung infiltrates: 1.32 (1.1–1.59); Point = 2 Chest X-ray - Other abnormality: 0.66 (0.3–0.98); Point = 2
Huang [20]	All suspected patients at admission	RT-PCR test	71	Demographics Comorbidities Vital signs Symptoms CT imaging Laboratory test	475	Logistic regression Score	Logistic regression: Epidemiological exposure histories (OR:13.32, 95%CI, 6.39–27.75), symptoms of weakness/fatigue at admission (OR:4.51, 95%CI, 1.70–11.96), heart rate less than 100 beat/min at admission (OR:3.80; 95%CI, 2.00–7.22), imaging characteristics of bilateral pneumonia (OR:3.60, 95%CI, 1.83–7.10), neutrophil count less than equal to 6.3 × 109 /L at admission (OR: 6.77, 95%CI, 2.52–18.19), eosinophil count less than equal to 0.02 × 109/L at admission (OR:3.14, 95%CI, 1.58–6.22), glucose more than equal to 6 mmol/L at admission (OR:2.43, 95%CI, 1.04–5.66), D-dimer more than equal to 0.5 mg/L at admission (OR:3.49; 95%CI, 1.22–9.96), and CRP less than 5 mg/L at admission (OR:3.83, 95%CI, 1.86–7.92) Score: Epidemiological exposure histories: 13 points Neutrophil count, × 109/L, ≤6.3: 7 points Weakness/fatigue: 5 points Bilateral pneumonia: 4 points Heart rate (beat/min), < 100: 4 points CRP, mg/L, < 5: 4 points Eosinophil count, × 109/L, ≤0.02: 3 points D-dimer, mg/L, ≥0.5: 3 points Glucose, mmol/L, ≥6: 2 points
Kurstjens [21]	Overcrowding of emergency departments	RT-PCR test	8	Demographics Laboratory test CT imaging	Model population: 375 Validation population: 592	Score	Age: ≤75: 0 points, 76–79: 1 point, 80+: 2 points Sex: Female: 0 point, Male: 1 point CRP, mg/L: 0–9: 0 point, 10–14: 1 point, 15–38: 2 points, 39–69: 3 points, 70–193: 2 points, 194–303: 1 point, 304+: 0 point Ferritin, μg/L: ≤15: − 1 point, 16–179: 0 point, 180–301: 1 point, 302–538: 2 points, ≥539: 3 points LDH, U/L: ≤257: 0 point, 258–265: 1 point, 266–397: 2 points, ≥398: 3 points ALC, 10^9/L: ≤1.2: 1 point, ≥1.3: 0 point ANC, 10^9/L: ≤5.1: 0 point, 5.2–7.9: − 1 point, 8.0–9.0: − 2 points, 9.1–10.3: − 3 points Chest X-ray: no infiltrate: 0 point, unilateral infiltrate: 1 point, bilateral infiltrate: 4 points
McDonald [22]	All patients greater than 18 years presenting to a single academic ED who were tested for COVID-19 during this index ED evaluation	RT-PCR test	44	Clinical data Demographics Symptoms Contact tracing Laboratory test	1026	Logistic regression Random forest XGBoost	Final logistic regression: Intercept: β = − 36 WBC: β = − 0.59, OR = 0.6 (0.4–0.8) Temperature: β = 0.44, OR = 1.6 (1.2–2.0) Known exposure: β = 1.51, OR = 4.5 (2.4–8.7) Positive chest X-ray: β = 1.69, OR = 5.4 (3–10)
Nakakubo [23]	Patients suspected of having COVID-19	RT-PCR test	31	Demographics Comorbidities Patient history Symptoms Respiratory failure Laboratory test CT imaging Differential diagnosis	131	Score	Blood test score WBC < 8000 (count/μL) = 1 point Eosinophil < 50 (count/μL) = 1 point Procalcitonin < 0.5 (ng/mL) and CRP ≥ 0.5 (mg/dL) = 1 point CT imaging score Ground glass opacity; with or without consolidation = 1 point Multipolar or bilateral lesions = 1 point Subpleural or lower lung dominant distribution = 1 point No atypical signs = 1 point Alternative diagnosis score (choose one) More likely other diagnosis = 0 point Hard to determine = 2 points More likely COVID-19 = 4 points Maximum 11 points 0–4 points: Low risk 5–7 points: Moderate risk 8–11: High risk
Plante [24]	Patients aged ≥20 years from 66 hospitals	RT-PCR test	29	Laboratory test	Training dataset: 12183 Sensitivity analysis dataset: 7842 External validation dataset: 172754	XGBoost	XGBoost model Eosinophils (%) 23.62 Calcium total (mg/dL) 16.85 AST (IU/L) 9.45 WBC (K/uL) 6.73 Basophils (%) 6.36 RDW (%) 5.13 RBC (m/uL) 5.11 Albumin (g/dL) 4.65 Bilirubin total (mg/dL) 4.30 MCV (fL) 3.98 MCH (pg) 3.49 Sodium (mEq/L) 3.29 Bicarbonate (mEq/L) 2.97 BUN (mg/dL) 2.40 Chloride (mEq/L) 1.68 Total score/100
Sung [16]	Patients who were admitted to the hospital from the emergency room to regular floors and tested for COVID-19	RT-PCR test	38	Demographics Medical history Contact tracing Laboratory test Symptoms Chest X ray imaging	Development cohort = 203 Validation cohort = 135	Logistic regression Score	Nursing facility residence: 9.63 (3.02–30.67), *p* < 0.001, Points: 10/2/3 Obesity: 2.93 (1.32–6.51), *p* = 0.008, Points: 3/1/1 Hypoxia: 3.52 (1.58–7.83), *p* = 0.002, Points: 4/1/1 Sick contact: 10.47 (2.67–41.04), *p* = 0.001, Points: 10/2/3 Constitutional symptom (fever, chills or myalgia): 2.31 (1.05–5.10), *p* = 0.038, Points: 2/1/1 Respiratory symptom (cough or shortness of breath): 4.36 (1.73–10.98), *p* = 0.002, Points: 4/1/1 Gastrointestinal symptom (nausea, vomiting or diarrhea): 4.11 (1.59–10.64), *p* = 0.004, Points: 4/1/1 Leukocytosis: 0.33 (0.12–0.90), *p* = 0.030, Points: − 3/− 1/− 1
Tordjman [14]	Subjects with clinical suspicion of SARS-CoV-2 infection	Both RT-PCR test and CT-Scan	31	Demographics Comorbidities Symptoms Laboratory test	Derivation cohort: 100 Validation cohort: 300	XGBoost	Logistic regression: Basophils < 4G/L: β = 1.66, OR = 5.27, *p*-value = 0.03 Eosinophils < 0.06 G/L: β = 1.98, OR = 7.23, *p*-value = 0.01 Lymphocytes < 1.3 G/L: β = 2.54, OR = 12.72, *p*-value = 0.001 Neutrophils <5G/L: β = 2.10, OR = 8.17, *p*-value = 0.006 Score: Eosinophils < 0.06 G/L: 1 point Lymphocytes < 1.3 G/L: 2 points Neutrophils <5G/L: 1 point Basophils < 0.04 G/L: 1 point Score, maximum 5 points: 0–1 - > low probability, 2–3: intermediate probability, ≥ 4 points
Vieceli [15]	Patients aged 18 or older admitted to hospital due to suspected COVID-19	RT-PCR test	43	Clinical data Laboratory test X ray imaging	100	Logistic regression	Logistic regression: Leukocyte count < 7.7 × 103 mm^3^: OR(CI) = 17.63 (3.68–84.7), *p*-value < 0.001 LDH > 273 U/L: OR(CI) = 5.42 (1.18–24.7), *p*-value = 0.03 Any chest radiographic abnormality: OR(CI) =27.8 (2.5–309.1), *p*-value = 0.007 Score: Two points for LDH > 273 U/L, three for leukocyte count ≤7.7 × 103 per mm^3^ and four for any chest radiography abnormality. A result ≥5 points was considered positive.

*Abbreviations*: *ALC* Absolute Lymphocyte Count, *ANC* Absolute Neutrophil Count, *AST* ASpartate aminotransferase, *BUN* Blood Urea Nitrogen, *CAD* Coronary Artery Disease, *CI* Confidence Interval, *CRP* C-Reactive Protein, *CT-scan/imaging* Computed Tomography-scan/imaging, *ED* Emergency Department, *LDH* Lactate DeHydrogenase, *MCH* Mean Corpuscular Hemoglobin, *MCV* Mean Corpuscular Volume, *OR* Odds Ratio, *qSOFA* quick Sepsis-related Organ Failure Assessment, *RBC* Red Blood Cells, *RDW* Red cells Distribution Width, *RT-PCR* reverse-transcription polymerase chain reaction, *SE* Standard Error, *VAS* Visual Analogic Scale, *WBC* White Blood Cells

For all the models developed, the gold standard was the RT-PCR (the study by Tordjam et al. [14] added also CT-scan as a gold standard). Numerous predictors of COVID-19 disease were candidate for the final model: at minimum 8 candidate predictors for the model of Kurstjens et al. [21], with a maximum of 71 in the study of Huang et al. [20]. The sample size varied from one study to another (i.e., from 100 participants for the studies of Bar et al. [13] and Vieceli et al. [15] to 172,754 for the study of Plante et al. [24]). The research team of Plante [24] was the only one to proceed to an external validation.

Most of the models were developed using logistic regressions. From this logistic regression, some developed a score from which it was possible to highlight the increased probability of suffering from COVID-19. Models such as XGBoost [14, 22, 24], random forest [22] and machine learning [17] were also applied. Three models did not offer the possibility to be quickly and easily applicable by the clinician, because not presenting scores: Bar *et al* [13], Fink *et al* [18], and McDonald *et al* [22], which limits the applicability of these models in practice.

The predictors proposed and analyzed by the different researchers were very diverse; however, we can observe a certain recurrence for certain predictors. The presence of fever appeared in 7 models, the blood value of eosinophils in 6 models, and CRP in 5 models. Four studies inserted comorbidities, gender (male) or chest X-ray as a predictor in their models. Finally, age, cough, WBC were significant predictors in three out of 13 studies and lymphocytes was present in two out of the 13 studies.

Moreover, to distinguish predictors directly available in an acute phase and in a non-acute phase is essential. Predictors directly available during the clinical examination are the most widely used: exposure risk, clinical signs, symptoms, clinical history, socio-demographic data, comorbidities, vital signs, contact tracing, respiratory failure, differential diagnosis. However, laboratory test and imaging need a waiting period to obtain results. All the models presented here mixed both type of predictors with the exception of Callejon-Leblic *et al* [17] which require only demographic and clinical data as well as symptoms.

While all models appeared to be successful in identifying patients at high risk for COVID-19, their performances were not similar. A summary of these is presented in Table 4.

**Table 4:** Diagnostic performance of the different included models in the systematic review of prediction models to diagnose COVID-19 from the start of the epidemic to March 2021

Reference	Classification measure	Discrimination measure
Aldobyany [12]	Score 4 sensitivity (Se), specificity (Sp), positive predictive value (PPV), and negative predictive value (NPV) were 65.9, 49.1, 28.8, and 82.1%, respectively. Score 5 sensitivity, specificity, positive predictive value, and negative predictive value are 64, 55.7, 31.1, and 83.2%, respectively.	The receiver operating characteristics ROC of COVID-19 respiratory triage score was above the line of no predictive value with an area under the ROC curve (AUROC) value of 0.60 (95% CI: 0.57–0.64).
Bar [13]	Sensitivity, 97% (83–100%); specificity, 62% (50–74%); PPV, 54% (41–98%); and NPV, 98% (88–99%)	AUROC for final logistic model: 0.82 (0.75–0.90).
Callejon-Leblic [17]	Sensitivity = 72 (69–75) % Specificity = 84 (82–87) % PPV = 83 (80–85) % NPV = 74 (71–77) %	AUROC = 0.78 (0.72–0.83)
Fink [18]	Using a cut-off threshold of 2 for the risk score, the diagnostic prediction model has a sensitivity of 78.1% and specificity of 86.8%. At COVID-19 prevalence of 85%, the diagnostic prediction model has a PPV of 95.1% and NPV of 36.0%. At COVID-19 prevalence of 10%, the PPV falls to 28.1% and NPV rises to 96.5%.	AUROC 0.8535 (95% CI (0.8121–0.8950). The optimism-corrected AUROC was 0.8465 (95% CI 0.7814–0.9038). The model performed comparably well for patients aged less than 80 years (AUROC 0.8736, 95% CI 0.8291–0.9181) and greater than 80 years (AUROC 0.8364, 95% CI 0.7492–0.9236).
Gupta-Wright [19]	Score threshold (< 4) Sensitivity = 26.6% Specificity = 96.6% PPV = 89% NPV = 56% Score threshold (> 9) Sensitivity = 37% Specificity = 96.1% PPV = 90.1% NPV = 61.2%	AUROC = 0.83 (0.82–0.85) for the model AUROC = 0.83 (0.81–0.84) for the score AUROC = 0.82 (0.80–0.84) for the bootstrapping
Huang [20]	A cut-off value of 20: specificity: 86.6%; sensitivity: 81.3%	A cutoff value of 20: AUROC was 0.921 (95%CI: 0.896–0.945, *P* < .01)
Kurstjens [21]	According to cutoffs value: 2, Se: 98% (0.96–0.99), Sp: 42%(0.35–0.49), True/False Negative: 83/7 3, Se: 98% (0.95–0.99), Sp: 53%(0.46–0.60), True/False Negative: 105/10 4, Se: 96% (0.94–0.98), Sp: 63%(0.56–0.70), True/False Negative: 125/15 5, Se: 94% (0.91–0.96), Sp: 72%(0.66–0.78), True/False Negative: 144/25 9, Se: 78% (0.73–0.82), Sp: 89%(0.84–0.93), True/False Negative: 305/22 10, Se: 68% (0.63–0.72), Sp: 92%(0.87–0.95), True/False Negative: 267/17 11, Se: 56% (0.51–0.61), Sp: 95%(0.90–0.97), True/False Negative: 219/11 12, Se: 45% (0.40–0.50), Sp: 97%(0.94–0.99), True/False Negative: 177/6	Model population: AUROC: 0.94 (95% CI 0.91–0.96) Validation population: 0.91 (95% CI 0.89–0.94)
McDonald [22]	Logistic regression: Se:97 (83–100) %, Sp: 69(62–75) %, PPV: 29 (20–36) %, NPV: 99(96–100) % Random forest: Se: 97 (83–100), Sp: 50 (43–57) %, PPV: 20 (14–28) %, NVP: 99 (95–100) % XGBoost: Se: 97 (83–100) %, Sp: 54 (47–61) %, PPV: 22 (15–30) %	Logistic regression: AUROC = 0.89(0.84–0.94) Random forest: AUROC = 0.86 (0.79–0.92) XGBoost: AUROC =0.85 (0.79–0.91)
Nakakubo [23]	Not reported	Not reported
Plante [24]	Score cutoff 1: Se: 95.9%, Sp: 41.7%, likelihood ratio: 0.099 Score cutoff 2: Se: 92.6%, Sp: 60.0%, likelihood ratio: 0.124 Score cutoff 5: Se: 85.5%, Sp: 78.5%, likelihood ratio: 0.185 Score cutoff 10: Se: 79.4%, Sp: 87.6%, likelihood ratio: 0.235	AUROC Training: 0.91 (0.90–0.92) External validation: 0.91 (0.90–0.92) Sensitivity analysis 0.89 (0.88–0.90)
Sung [16]	Development cohort (Risk score > =3) Sensitivity = 85.1% Specificity = 75% PPV = 71.8% NPV = 87% Validation cohort Sensitivity = 79.6% Specificity = 70.9% PPV = 60.9% NPV = 85.9%	Development cohort Model 1: AUROC = 0.87 (0.83–0.92), Model 2: AUROC = 0.87 (0.83–0.92), Model 3: AUROC = 0.87 (0.82–0.92) Validation cohort Model 1: AUROC = 0.85 (0.78–0.92), Model 2: AUROC = 0.83 (0.76–0.90), Model 3: AUROC = 0.85 (0.78–0.92)
Tordjman [14]	PARIS score: 0: sensitivity = 100%, specificity = 0% 1: sensitivity = 100%, specificity = 28% 2: sensitivity = 99%, specificity = 53% 3: sensitivity = 92%, specificity = 72% 4: sensitivity =79%, specificity = 90% 5: sensitivity = 38%, specificity = 99%	Validation cohort: AUROC = 0.889, for score ≥ 4 points Derivation cohort: AUROC = 0·921; STD = 0·027; CI = [0·867–0·974]
Vieceli [15]	Score: 96% of sensitivity, 73.5% of specificity	Before bootstrapping: AUROC of 0.847 (95% CI 0.77–0.92) After bootstrapping: AUROC of 0.827 (95% CI 0.75–0.90)

The sensitivity and specificity varied greatly between the different models but also according to the possible differences in score results offered by certain models. The Kurstjens et al. [21] model seemed to offer the best sensitivity (98%), while the Gupta-Wright et al. [19] model seems to offer the best specificity (96.1%).

All the models performed well to discriminate people with or without COVID-19 (all AUROCs> 0.500). The lowest AUROC was observed for the model of Aldobyany [12] (i.e., an AUROC of 0.600) and the best AUROC was that of the model of Kurstjens et al. [21] (i.e., an AUROC of 0.940).

**Fig. 2:**
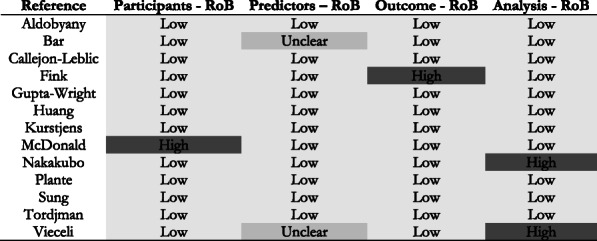
Risk of Bias for each individual study in the systematic review of prediction models to diagnose COVID-19 from the start of the epidemic to March 2021 using the PROBAST tool

RoB has been assessed by two independent reviewers for each individual studies (see Fig. 2). In general, the RoB was considered low. However, participants selection was at high RoB in one study (McDonald et al. [22]) due to difficulties to estimate the appropriateness of the inclusion and exclusion criteria and the data source. High RoB regarding the outcome was estimated for the model of Fink et al. [18] because of information about the outcome were lacking. Finally, analysis could engender a RoB in the studies of Nakakubo et al. [23] and Vieceli et al. [15] because they did not respond to some pre-determined criteria (e.g., statistical selection of predictors, overfitting in model performance not accounted, consideration of all enrolled participants,…). In Fig. 2, it was considered unclear when the paper did not provide enough information to assess the RoB [13, 15].

## Discussion

### Model identification

Our systematic review of the literature identified 13 studies presenting models for predicting patients at high risks of COVID-19 from those likely not presenting with COVID-19.When limiting the target population to COVID-19 suspected patients in hospital settings in contrast of the study of Wynants et al. [6], the results indicated that the prominent predictors were similar to those found in the study of the whole type of population [6]., namely socio-demographics (age and gender), clinical symptoms, vital signs, laboratory or biological tests. The recurrent predictors respective to their appearance in the studies were fever (7), eosinophil (6), CRP (5), male gender (4), chest X-ray (4), (older) age (3), cough (3), WBC (3), and lymphocytes (2). The inclusion of comorbidities was observed in four out of 13 studies. Yet, comorbidities were of little predictive value for COVID-19 positivity in the present study and in Wynants et al. (2020); only obesity, chronic renal failure and CAD/heart failure were found to be significant predictors. In this regard, Vieceli et al. [15] postulated that patients with more comorbidities could have less exposure risk due to their low travelling possibilities.

### Model comparison

The authors employed various methods to develop their models such as logistic regression, score, XGBoost, machine learning with logistic regression being the most employed method. The number of predictors differed substantially, ranging from 8 to 71 variables as per study. Despite the unneglectable false-negative rate, RT-PCR was adopted as the gold standard in all included studies, except in Tordjman et al. [14] where both RT-PCR and CT-scan were used.

The final resulted models were either simple with as few variables as four (McDonald et al. [22]) or complex with 15 variables (Plante et al. [22]). To advance practical uses, authors also proposed scores [12, 16, 19–21, 23] which eased the calculation process and facilitate clinical use. The nature of the predictors varied across studies (i.e., the included models incorporated different combinations of socio-demographics (age and gender), clinical symptoms, vital signs, laboratory or biological tests). This has an implication in terms of applicability in clinical practices. If the models are to be used to triage patients at first admission, those variables that can be immediately collected such as age, gender, symptoms or tracing contact can be more useful than laboratory or biological tests. Otherwise, the latter are more appropriate for research purposes aimed at the medical/biological aspects of the COVID-19.

When the same independent variables were used, it was noticed that there were also differences in the measurement and threshold used. For example, the cut-off values for age and biological indicators like lymphocytes or CRP (mg/L) varied across models. In this respect, the generalization of the models to a different context is not plausible given that standardized thresholds are not available. Furthermore, in some cases, the collected variables were country-specific like the qSOFA (Bar et al. [11]) and cannot be obtained if the model is to be put into use in a setting other than the research context.

With the discussed heterogeneity in terms of variable selection and measurement, a straight-forward implementation of a given model included in this systematic review is not recommended. Although we have applied rigorous criteria in terms of (adult) population, (hospital) setting, and design and population, i.e., consisting of only COVID-19 suspected patients and removing interventional studies, the confidence in specifying a model deemed most promising for clinical use requires further investigation. However, on the basis of the findings, future studies could make use of the most recurrent significant predictors to further develop or refine existing prediction models.

### Model development, performance, and evaluation

Among the included studies, the sample size ranged from less than 200 (Bar et al. [11], Nakakubo et al. [23], and Vieceli et al. [15]) to as high as 192,779 (Plante et al. [22]). In the case of small or medium sample size, the justification whether the sample size was adequate for the tested prediction model was not reported. All studies made use of convenience sampling rather than attempting to realize more rigorous method such as stratified sampling techniques.

Most studies were conducted at a single site or institution, except those from Gupta-Wright et al. [17], and Plante et al. [22]. The latter was a large-scale study which involved data of blood tests from 66 US hospitals. When validation was taken into account, all of the studies (Kurstjens et al. [19], Tordjman et al. [23], and Sung et al. [14], Gupta-Wright et al. [17]) employed a development and validation cohorts or bootstrapping, hence internal validation was the most frequently used method. An exception was that of Plante et al. [22] where external validation was feasible due to scale of the study. The finding echoes the observation from Wynant et al. such that overfitting and low predictive power in a new context might be a concern. In this regard, future studies can make use of recommendations for sample size estimation and validation from Riley et al. to achieve more robust predictive models.

To evaluate the model, all studies reported sensitivity and specificity and/or NPV and PPV as the main discriminative indicators, except that from Nakakubo et al. [21] in which no such information was available. No calibration plot was reported in the included studies, which limits insights into the extent to which the developed models are calibrated. All of these models are effective in discriminating patients according to the probability of COVID-19 infection (all AUROCs> 0.500). Therefore, to an extent, these models could demonstrate their discriminative effectiveness.

Focusing on the target population of COVID-19 suspected adult patients in hospital settings only, the systematic review attempted to select studies with a clear description of the patient characteristics and context. Using this criteria, one study (McDonald et al. [20]) was deemed as pertaining a bias in the participant selection because, due to the decision of the health care systems, no asymptomatic testing was performed. The bias in terms of predictors was mostly low, except in Bar et al. [11] and Vieceli et al. [24] in which the risk was unclear due to the exclusion of cases with low quality ultrasound scan. Low bias regarding the outcome was observed in most studies, which was expected due to the use of a common gold standard of RT-PCR. In Fink et al. [13], there was a potential risk because only the results obtained within 72 h were considered eligible. As regards data analysis methods, the models from Nakakubo et al. [21] and Vieceli et al. [24] were more likely to be biased. In the former, the authors assigned arbitrary risk scores based on the univariate results from Fisher exact test, t-test or Mann-Whitney test. In the latter, the bias could be attributable to the small sample size (*n* = 100) and a high number of predictors of 43.

### Implications for research and practices

The systematic review has identified 13 models worth considering when suspected COVID-19 adult patients in a hospital setting are the target population. Based on the findings, the following implications could be helpful for research and practices.

First of all, the findings revealed recurrent significant predictors which are in accordance with the latest (and living) systematic literature by Wynants et al. When only the positivity of COVID-19 is of interest rather than disease progression or severity, comorbidities might be less important than socio-demographics (age and gender), clinical symptoms, vital signs, laboratory or biological tests. Therefore, future studies could rely on the identified groups of predictors to refine COVID-19 prediction models and further clarify the role of comorbidities regarding COVID-19 infection probabilities.

Second, as can be seen, there are different groups of variables collected in the presented models in this systematic review. Variables could be immediately collected at first screening included socio-demographics (age and gender), clinical symptoms, or tracing contact while others such as vital signs, laboratory or biological tests and chest X-rays are only available at a later stage of the admission process. Therefore, when testing facilities are limited and early screening is essential to adequately and rapidly triage patient flows to prevent nosocomial transmission, the clinicians could make use of age and gender as well as the significant clinical symptoms identified at first screening. In case an immediate level of risk is resulted rather than a high or a low-risk category, perhaps it would be more favored to promote over-triage and subsequently perform further examinations to confirm COVID-19 positivity. In so doing, either testing resources could be effectively employed or false negatives could be minimized to the least extent.

Third, a narrative comparison of the 13 models revealed more heterogeneity in terms of research design, model complexity, variable selection and measurement, model development methods and statistical reporting. While posing challenges for the applicability of these predictive models in practice, future research could capitalize on these existing models to better develop or refine predictive models in the specific hospital setting. Another research venture is to examine the extent to which the potential models, i.e., those with adequate sample sizes and sound methodologies, could yield comparable predictive results using agreement indexes. External validation of the potential models using standardized and multi-centered datasets is definitely valuable to advance our knowledge of the critical predictors of COVID-19 infection. In so doing, measures to mitigate community and nosocomial infections could be more efficient.

Fourth, despite the fact that biases were inherent in all models included in this systematic review, it is believed that the biases are sometimes beyond scientific decisions, particularly when they were developed in such a pandemic context. In other words, the complexity of the viral mutation, the pressure of the healthcare systems when facing the exponential growth of the viral transmission, and the availability of testing infrastructures have induced certain constraints for efforts to ensure research integrity. Therefore, instead of viewing the biases as limitations regarding model application in practice, it is advised that policy makers, researchers, and clinicians could interpret these models as potential models in a given setting and constraints. Methodologically speaking, the application of any model in practice should undergo a thorough investigation and pilot testing phase. In this respect, findings from the present systematic review are helpful to initiate such investigations.

Our study encounters certain limitations which need to be considered, hence entailing a cautious interpretation of the results. First, two databases have been searched (Scopus and MEDLINE); however, other databases search (e.g., EMBASE) could have been optimal although not possible in our case due to logistical constraints. Second, we limited our search only to published studies. Nonetheless, in the context of COVID-19, we have found it more appropriate to focus on peer-reviewed studies. Our work is set in time when we suspect that many studies could be published in the future. To do this, we have to carry out the most recent update possible. To counter this time constraint, carrying out a living systematic review would be optimal.

## Conclusion

The present systematic review yielded 13 predictive models of COVID-19 for suspected adult patients admitted to a health care department. Using rigorous inclusion criteria, the selected models informed and confirmed our knowledge of the most important predictors of COVID-19. Despite the inherent biases that were inevitable, the 13 models could demonstrate their effectiveness in predicting COVID-19 positive cases employing indicators like sensitivity, specificity, PPV, NPV, and AUROC. Potential models among the given 13 models might be selected depending on the objective, the target population, and the implementation context in terms of human resources and testing infrastructure, which should be subject to further testing and refining. Future research could reliably base on the findings from this systematic review to advance current knowledge of the significant predictors of COVID-19 using RT-PCR as the gold standard across different contexts.

## Supplementary Information

**Additional file 1: Supplementary material.** Search strategy and listing of excluded studies at the full-text stage.

## Data Availability

Provided as supplementary material.

## Abbreviations

ALC: Absolute Lymphocyte Count
ANC: Absolute Neutrophil Count
AST: ASpartate aminoTransferase
AUROC: Area Under the ROC curve
BUN: Blood Urea Nitrogen
CAD: Coronary Artery Disease
CHARMS: CHecklist for critical Appraisal and data extraction for systematic Reviews of prediction Modelling Studies
CI: Confidence Interval
COVID-19: COronaVIrus Disease 2019
CRP: C-Reactive Protein
CT-scan/imaging: Computed Tomography-scan/imaging
ED: Emergency Department
LDH: Lactate DeHydrogenase
MCH: Mean Corpuscular Hemoglobin
MCV: Mean Corpuscular Volume
NPV: Negative Predictive Value
OR: Odds Ratio
PPV: Positive Predictive Value
PRISMA: Preferred Reporting Items for Systematic Reviews and Meta-analyses
PROBAST: Prediction model study Risk Of Bias ASsessment Tool
qSOFA: quick Sepsis-related Organ Failure Assessment
RBC: Red Blood Cells
RDW: Red cells Distribution Width
RoB: Risk of Bias
RT-PCR: Reverse Transcription-Polymerase Chain Reaction
SARS: Severe Acute Respiratory Syndrome
SARS-CoV-2: Severe Acute Respiratory Syndrome CoronaVirus 2
SE: Standard Error
VAS: Visual Analogic Scale
WBC: White Blood Cells
