# Supplementary material for: A systematic review of prediction models to diagnose COVID-19 in adults admitted to healthcare centers
Source: Arch Public Health. 2021 Jun 18;79:105. doi: 10.1186/s13690-021-00630-3 (PMC8211973; doi:10.1186/s13690-021-00630-3)
Supplement: Supplementary file 1 — Additional file 1: Supplementary material. Search strategy and listing of excluded studies at the full-text stage. [file 13690_2021_630_MOESM1_ESM.docx]

Supplementary Material

Database: **Ovid MEDLINE(**R) and Epub Ahead of Print, In-Process & Other Non-Indexed Citations, Daily and Versions(R) <1946 to January 04, 2021>

Search Strategy:

--------------------------------------------------------------------------------

1 COVID-19.ti,ab,kf.

2 COVID-2019.ti,ab,kf.

3 "severe acute respiratory syndrome coronavirus 2".ti,ab,kf.

4 2019-nCoV.ti,ab,kf.

5 SARS-CoV-2.ti,ab,kf.

6 2019nCoV.ti,ab,kf.

7 ((Novel adj1 corona*) and "2019").ti,ab,kf.

8 Coronavirus/

9 "2019".ti,ab,kf.

10 or/1-7

11 8 and 9

12 10 or 11

13 ((predict* or prognos* or equation) adj2 (model* or rule* or index* or score* or tool*)).ti,ab,kf.

14 ((risk* or probabilit* or causal* or factor*) adj2 (predict* or decision* or score* or assess* or apparais* or equation* or calculate* or tool*)).ti,ab,kf.

15 13 or 14

16 exp Diagnosis/

17 (diagnos* or detect*).ti,ab,kf.

18 exp "Sensitivity and Specificity"/

19 (sensitiv* or specific*).ti,ab,kf.

20 (roc adj2 (analys* or curve*)).ti,ab,kf.

21 "receiver operating characteristic".ti,ab,kf.

22 ((pre-test or pretest) adj2 probabili*).ti,ab,kf.

23 or/16-22

24 12 and 15 and 23

25 exp animals/ not humans.sh.

26 (exp infant/ or exp child/ or adolescent/) not exp adult/

27 24 not 25 not 26

28 limit 27 to english language

29 limit 28 to dt=20200501-20210101 [May 1st, 2020 to January 4th 2021] (update February 26^th^)

**SCOPUS**

( ( ( TITLE-ABS-KEY ( COVID-19 ) )  OR  ( TITLE-ABS-KEY ( COVID-2019 ) )  OR  ( TITLE-ABS-KEY ( "severe acute respiratory syndrome coronavirus 2" ) )  OR  ( TITLE-ABS-KEY ( 2019-ncov ) )  OR  ( TITLE-ABS-KEY ( sars-cov-2 ) )  OR  ( TITLE-ABS-KEY ( 2019ncov ) )  OR  ( TITLE-ABS-KEY ( ( ( novel  W/1  corona* )  AND  "2019" ) ) ) )  AND  ( ( TITLE-ABS-KEY ( ( ( predicti*  OR  prognos*  OR  equation )  W/2  ( model  OR  rule  OR  index  OR  score  OR  tool ) ) ) )  OR  ( TITLE-ABS-KEY ( ( ( risk  OR  probability  OR  causal*  OR  factor )  W/2  ( predict*  OR  decision  OR  score  OR  assess*  OR  apparais*  OR  equation  OR  calculate*  OR  tool ) ) ) ) ) )  AND  ( ( TITLE-ABS-KEY ( ( diagnos*  OR  detect*  OR  sentitivit*  OR  specificit*  OR  roc  OR  "receiver operating characteristic" ) ) )  OR  ( TITLE-ABS-KEY ( ( ( pretest  OR  pre-test )  W/2  probabilit* ) ) ) )  AND  ( LIMIT-TO ( PUBYEAR ,  2021 )  OR  LIMIT-TO ( PUBYEAR ,  2020 ) )  AND  ( LIMIT-TO ( LANGUAGE ,  "English" ) )

| Supplementary material – Listing of excluded studies at the full-text stage | | | |
| --- | --- | --- | --- |
| **First author, date** | **Title** | **Journal** | **Reason of exclusion** |
| Raberahona 2021 | Clinical and epidemiological features discriminating confirmed COVID-19 patients from SARS-CoV-2 negative patients at screening centres in Madagascar |  | Additional data not provided |
| Zoabi 2021 | Machine learning-based prediction of COVID-19 diagnosis based on symptoms. | NPJ digital medicine | Wrong outcome |
| Hermans 2020 | Chest CT for triage during COVID-19 on the emergency department: myth or truth?. | Emergency radiology | Additional data not provided |
| Delafiori 2021 | Covid-19 Automated Diagnosis and Risk Assessment through Metabolomics and Machine Learning. | Analytical chemistry | Wrong outcome |
| Joshi 2020 | A predictive tool for identification of SARS-CoV-2 PCR-negative emergency department patients using routine test results. | Journal of clinical virology: the official publication of the Pan American Society for Clinical Virology | Wrong study design |
| Liu 2020 | A Two-Dimensional Sparse Matrix Profile DenseNet for COVID-19 Diagnosis Using Chest CT Images |  | Wrong population |
| Oshman 2020 | Whom Should We Test for COVID-19? Performance of a Symptom and Risk Factor Questionnaire on COVID-19 Test Results and Patient Outcomes in an Immediate Care Setting. | Journal of primary care & community health | Additional data not provided |
| Jehi 2020 | Individualizing Risk Prediction for Positive Coronavirus Disease 2019 Testing: Results From 11,672 Patients. | Chest | Wrong population |
| Langer 2020 | Development of machine learning models to predict RT-PCR results for severe acute respiratory syndrome coronavirus 2 (SARS-CoV-2) in patients with influenza-like symptoms using only basic clinical data. | Scandinavian journal of trauma, resuscitation and emergency medicine | Additional data not provided |
| Chen 2020 | A new rapid screening program based on risk scores for COVID-19 patients. | Internal and emergency medicine | Wrong population |
| Kimhofer 2020 | Integrative Modeling of Quantitative Plasma Lipoprotein, Metabolic, and Amino Acid Data Reveals a Multiorgan Pathological Signature of SARS-CoV-2 Infection. | Journal of proteome research | Wrong population |
| Ng 2020 | Development and validation of risk prediction models for COVID-19 positivity in a hospital setting. | International journal of infectious diseases: IJID : official publication of the International Society for Infectious Diseases | Wrong population |
| Mao 2020 | Assessing risk factors for SARS-CoV-2 infection in patients presenting with symptoms in Shanghai, China: a multicentre, observational cohort study. | The Lancet. Digital health | Wrong outcome |
| Raberahona 2020 | Clinical and epidemiological features discriminating confirmed COVID-19 patients from SARS-CoV-2 negative patients at screening centres in Madagascar. | International journal of infectious diseases: IJID: official publication of the International Society for Infectious Diseases | Wrong outcome |
| Avila 2020 | Hemogram data as a tool for decision-making in COVID-19 management: applications to resource scarcity scenarios. | PeerJ | Preprint |
| Horvath 2020 | Comparison of clinical characteristics of patients with pandemic SARS-CoV-2-related and community-acquired pneumonias in Hungary - a pilot historical case-control study. | GeroScience | Wrong population |
| Fang 2020 | Radiomics nomogram for the prediction of 2019 novel coronavirus pneumonia caused by SARS-CoV-2. | European radiology | Wrong population |
| vanWalraven 2020 | Derivation and Internal Validation of a Model to Predict the Probability of Severe Acute Respiratory Syndrome Coronavirus-2 Infection in Community People. | Journal of general internal medicine | Wrong study design |
| Trubiano 2020 | COVID-MATCH65-A prospectively derived clinical decision rule for severe acute respiratory syndrome coronavirus 2. | PloS one | Wrong population |
| Banerjee 2020 | Use of Machine Learning and Artificial Intelligence to predict SARS-CoV-2 infection from Full Blood Counts in a population. | International immunopharmacology | Wrong outcome |
| LopezdelaIglesia 2020 | Predictive factors of COVID-19 in patients with negative RT-qPCR. | Semergen | Wrong outcome |
| Smith 2020 | A Symptom-Based Rule for Diagnosis of COVID-19. | SN comprehensive clinical medicine | Wrong population |
| Wu 2020 | A prediction model of outcome of SARS-CoV-2 pneumonia based on laboratory findings. | Scientific reports | Wrong population |
| Lee 2020 | Self-reported anosmia and dysgeusia as key symptoms of coronavirus disease 2019 |  | Wrong study design |
| O'Reilly 2020 | Epidemiology and clinical features of emergency department patients with suspected COVID-19: Results from the first month of the COVID-19 Emergency Department Quality Improvement Project (COVED-2). | Emergency medicine Australasia : EMA | Wrong outcome |
| Muhammad 2021 | Supervised Machine Learning Models for Prediction of COVID-19 Infection using Epidemiology Dataset. | SN computer science | Wrong study design |
| Wee 2020 | Containing COVID-19 in the Emergency Department: The Role of Improved Case Detection and Segregation of Suspect Cases |  | Wrong outcome |
| Woolford 2020 | COVID-19 and associations with frailty and multimorbidity: a prospective analysis of UK Biobank participants |  | Wrong population |
| Liu 2020 | Risk factors associated with COVID-19 infection: a retrospective cohort study based on contacts tracing |  | Wrong population |
| Atkins 2020 | Preexisting Comorbidities Predicting COVID-19 and Mortality in the UK Biobank Community Cohort |  | Wrong outcome |
| Wei 2020 | A real-time robot-based auxiliary system for risk evaluation of COVID-19 infection |  | Wrong population |
| Somasekar 2020 | Machine Learning and Image Analysis Applications in the Fight against COVID-19 Pandemic: Datasets, Research Directions, Challenges and Opportunities. | Materials today. Proceedings | Wrong population |
| Abbas 2020 | Establishment of diagnostic protocols for covid-19 patients |  | Wrong outcome |
| CheAzemin 2020 | COVID-19 Deep Learning Prediction Model Using Publicly Available Radiologist-Adjudicated Chest X-Ray Images as Training Data: Preliminary Findings. | International journal of biomedical imaging | Wrong outcome |
| Lv 2020 | The prognostic value of general laboratory testing in patients with COVID-19 |  | Wrong population |
| Schwab 2020 | Clinical Predictive Models for COVID-19: Systematic Study. | Journal of medical Internet research | Wrong population |
| Zhang 2020 | Differences and prediction of imaging characteristics of COVID-19 and non-COVID-19 viral pneumonia: A multicenter study. | Medicine | Wrong outcome |
| Challener 2020 | Screening for COVID-19: Patient factors predicting positive PCR test. | Infection control and hospital epidemiology | Wrong outcome |
| Just 2020 | Risk factors for a positive SARS-CoV-2 PCR in patients with common cold symptoms in a primary care setting: a retrospective analysis based on a joint documentation standard |  | Wrong outcome |
| Albahri 2021 | Detection-based prioritization: Framework of multi-laboratory characteristics for asymptomatic COVID-19 carriers based on integrated EntropyTOPSIS methods |  | Wrong outcome |
| Bayat 2020 | A SARS-CoV-2 Prediction Model from Standard Laboratory Tests. | Clinical infectious diseases: an official publication of the Infectious Diseases Society of America | Wrong population |
| Tschoellitsch 2020 | Machine Learning Prediction of SARS-CoV-2 Polymerase Chain Reaction Results with Routine Blood Tests. | Laboratory medicine | Wrong outcome |
| Roland 2020 | Smell and taste symptom-based predictive model for COVID-19 diagnosis. | International forum of allergy & rhinology | Wrong population |
| Ma 2020 | Development and validation of a risk stratification model for screening suspected cases of COVID-19 in China. | Aging | Wrong population |
| Wei 2020 | A risk assessment system of COVID-19 based on Bayesian inference |  | Wrong population |
| Levenfus 2020 | Triage tool for suspected COVID-19 patients in the emergency room: AIFELL score. | The Brazilian journal of infectious diseases: an official publication of the Brazilian Society of Infectious Diseases | Wrong study design |
| Yang 2020 | Routine Laboratory Blood Tests Predict SARS-CoV-2 Infection Using Machine Learning. | Clinical chemistry | Wrong population |
| Sun 2020 | Epidemiological and clinical predictors of COVID-19 |  | Wrong population |
| Qin 2020 | A predictive model and scoring system combining clinical and CT characteristics for the diagnosis of COVID-19. | European radiology | Wrong population |
| Diaz-Quijano 2020 | A model to predict SARS-CoV-2 infection based on the first three-month surveillance data in Brazil. | Tropical medicine & international health: TM & IH | Wrong population |
| Liu 2020 | A COVID-19 Risk Assessment Decision Support System for General Practitioners: Design and Development Study. | Journal of medical Internet research | Wrong outcome |
| Ghazi 2020 | Predicting patients with false negative SARS-CoV-2 testing at hospital admission: A retrospective multi-center study. | medRxiv : the preprint server for health sciences | Preprint |
